# Isoleucine and valine regulate the BCAA antagonism by influencing insulin function in broiler chickens

**DOI:** 10.1186/s40104-025-01326-2

**Published:** 2026-02-11

**Authors:** Bin Wang, Xiaodan Zhang, Guang Li, Mingkun Gao, Yuqing Feng, Yong He, Yuming Guo

**Affiliations:** 1 State Key Laboratory of Animal Nutrition and Feeding, College of Animal Science and Technology, China Agricultural University, Beijing, 100193 China; 2 Shenyang Boeing Feed Company, Shenyang, 110141 China

**Keywords:** Antagonism, Branched-chain amino acids, Broiler chicken, Inflammatory factors, Insulin

## Abstract

**Background:**

The phenomenon where excessive activation of branched-chain amino acid (BCAA) degrading enzymes caused by high concentrations of leucine (Leu) leads to a decrease in the overall concentration of BCAA [including isoleucine (Ile) and valine (Val)] is called BCAA antagonism. Although this phenomenon has long been widely studied, the specific mechanism of its occurrence is still poorly understood. In this study, we investigated the specific mechanism by which Val and Ile alleviate the antagonistic effect caused by high concentrations of Leu through influencing insulin function. First, the ratios of Ile and Val in the low-protein diet were adjusted up and down by 15% to observe the metabolic status of broilers at the end of the experiment (the experiment period was from 0 to 42 d). Subsequently, the physiological and biochemical changes related to antagonism were determined using transcriptome and lipid metabolome analyses.

**Results:**

When fed with a high concentration of Leu, restricting Ile or supplementing Val can effectively alleviate antagonism. Under conditions of excessive dietary Val supplementation, insulin levels remained stable, whereas blood glucose levels increased (*P* < 0.05), and liver fat accumulated predominantly as ceramides rather than triglycerides, thereby disrupting the insulin-mediated phosphatidylinositol 3-kinase/protein kinase B signaling pathway (*P* < 0.05). Excessive dietary Ile promoted liver inflammation and interleukin-6 release (*P* < 0.05), which acted on the pancreas to enhance insulin secretion. Additionally, the glucagon content in the pancreas decreased (*P* < 0.05), while insulin and glucagon-like peptide-1 levels increased (*P* < 0.05).

**Conclusion:**

Supplementation of Val or restriction of Ile in low-protein diets could alleviate the BCAA antagonism caused by high Leu, which mainly achieved by influencing insulin function. These findings provide new insights in revealing the BCAA antagonism.

**Graphical Abstract:**

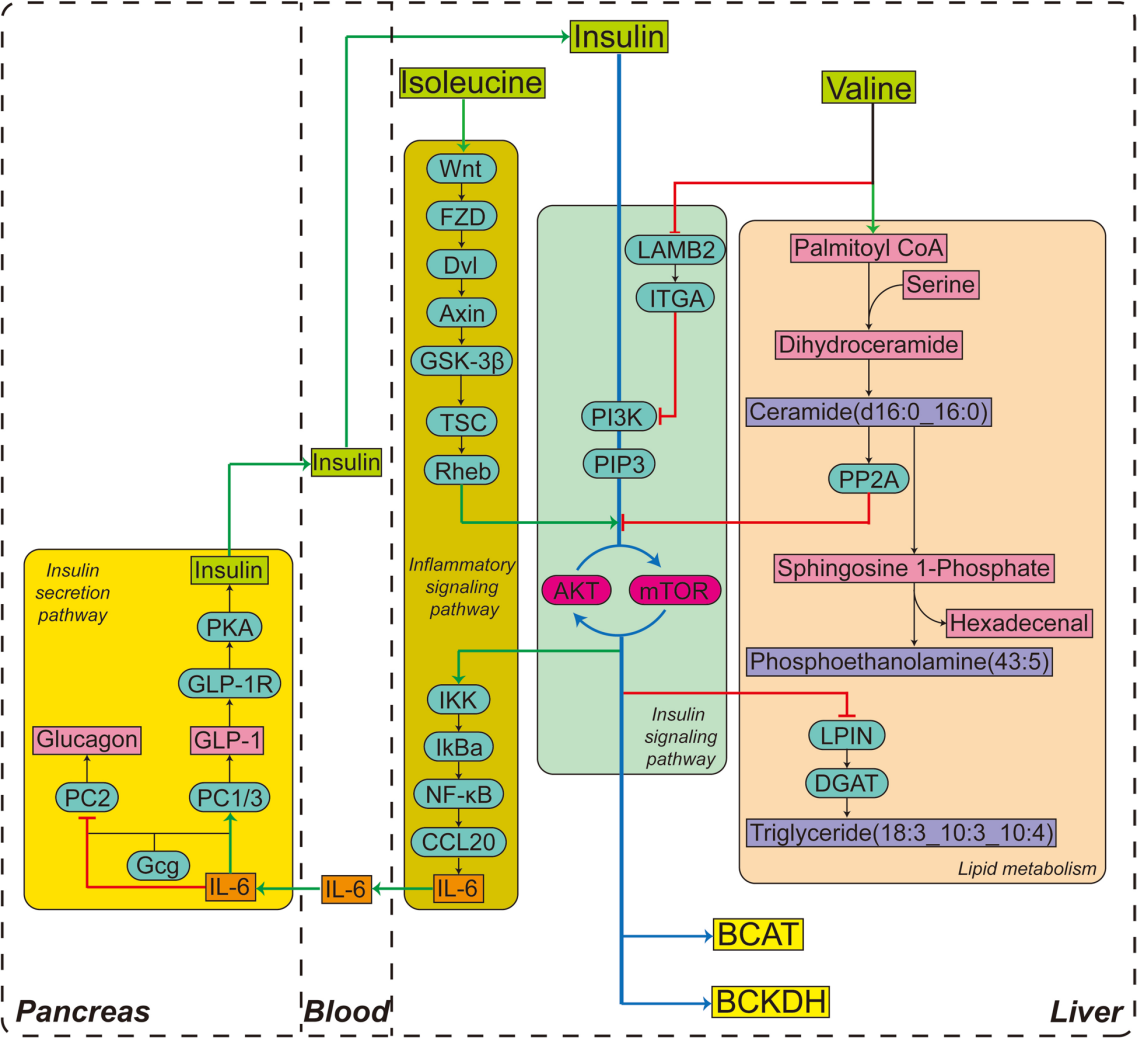

## Introduction

In poultry diets, branched-chain amino acid (BCAA) antagonism has become increasingly prominent with the widespread use of low-protein diets when dietary amino acids are not well balanced. Moreover, chickens are highly sensitive to fluctuations in dietary amino acids [1], which further increases the severity of this situation.

BCAAs, including leucine (Leu), isoleucine (Ile), and valine (Val), are essential amino acids and their antagonistic interactions have been well documented [2]. Leu, which is abundant in grains, plays a dominant role in antagonism, suggesting that the incidence of antagonism in low-protein diets is much higher than in normal-protein diets. In contrast, Ile and Val exhibit milder effects, with Val supplementation being more effective than Ile in mitigating the antagonistic effects of excessive dietary Leu [3]. To fully harness the benefits of a low-protein diet, it is crucial to understand the mechanisms underlying BCAA antagonism and develop strategies to regulate it, thereby ensuring adequate amino acid nutrition under this dietary regimen.

In recent years, an increasing number of studies have demonstrated that the concentration of BCAAs in blood circulation significantly increases under specific disease conditions, such as obesity, maple syrup urine disease, and type I and type II diabetes [4]. A study has identified blood BCAA levels as sensitive biomarkers for predicting diabetes onset [5]. Blood levels of BCAAs are directly linked to the activity of key enzymes involved in their catabolism, namely branched-chain aminotransferase (BCAT) and branched-chain alpha-keto dehydrogenase (BCKDH) [6]. Decreased research and impaired activity have been observed in animal models of obesity and type II diabetes [7]. In contrast, studies in healthy individuals revealed that blood BCAA levels significantly decrease following glucose infusion [8]. Although both experimental groups were hyperglycemic, there were marked differences in BCAA catabolism. This paradoxical observation suggests that there is a critical relationship between insulin and BCAA catabolism [9]. A rat model study further confirmed this correlation by showing that insulin infusion led to a rapid decline in blood BCAA levels and a significant increase in the expression and activity of hepatic catabolic enzymes, thereby highlighting the pivotal role of insulin in regulating BCAA metabolism [10]. These findings underscore the importance of insulin in understanding the antagonism among BCAAs.

The relationship between BCAA and insulin function is intricate and remains a subject of debate, with conflicting findings reported across studies. The primary controversy is the paradoxical dual effects of BCAA, which can enhance insulin sensitivity and induce insulin resistance [11, 12]. Leu interacts closely with mammalian target of rapamycin (mTOR), effectively promoting muscle protein synthesis, and exhibits synergistic effects with insulin [13]. In experiments involving the infusion of individual BCAA into the hypothalamus of hyperglycemic rats, Ile and Leu significantly reduced blood glucose levels [14], suggesting their potential roles in regulating glucose homeostasis and improving insulin sensitivity. Conversely, Val did not demonstrate similar benefits and was more likely to contribute to insulin resistance in mice fed a high-fat diet [15], indicating its possible role in fat metabolism. These observations highlight the distinct functional differences among the BCAA members. Despite their highly similar molecular structures, it is imperative to further investigate and elucidate the specific roles of each BCAA component.

Therefore, this study aimed to investigate the specific mechanisms through which Ile and Val alleviate antagonistic under conditions of leu excess, leveraging their distinct functional roles, and to demonstrate that both amino acids exert these effects by modulating insulin function.

## Materials and methods

### Animal feeding management and diets

A total of 300 male Arbor Acres broilers were used in experiment. Each treatment involved six replicates, with 10 broilers per cage. The rearing was divided into three stages: starter: 0–14 d; grower: 15–28 d; finisher: 29–42 d. The experimental diets were formulated based on the Poultry Feeding Standards of China (2004) [16], the normal protein diet in which were 23%, 21.5%, and 20%. On this basis, a 2% reduction in crude protein level was made to form the low-protein diet in this study. The protein levels in the low-protein diet were 21%, 19.5%, and 18%, respectively. The amino acid levels of other diets were adjusted according to the low-protein diet. The composition, nutritional levels, and corresponding amino acid ratios of the diets are shown in Table 1. In experiment, under the condition of high Leu concentration, the Val or Ile concentration was adjusted by 15% up and down to investigate the antagonistic response mechanism. All the birds were housed in cages with free access to feed and water. The lighting period in the poultry house was gradually reduced from 24 h/d to 20 h/d, and the temperature was gradually decreased from 35 to 22 °C. The immunization schedule was conducted according to the management manual, and the environmental conditions of the poultry house and health status of the broilers were monitored daily. All broiler experiments were conducted at the China Agricultural University Zhuozhou Experimental Base and approved by the Animal Welfare and Ethics Committee of China Agricultural University (AW61504202-1-2).

**Table 1:** Ingredients and nutrient composition of diets (as-fed basis)

Item	Starter					Grower					Finisher				
	LP	LPLV	LPHV	LPLI	LPHI	LP	LPLV	LPHV	LPLI	LPHI	LP	LPLV	LPHV	LPLI	LPHI
Corn (level 2, 7.8%), %	56.95	56.95	56.95	56.95	56.95	68.45	68.45	68.45	68.45	68.45	67.75	67.75	67.75	67.75	67.75
Soybean meal (level 3, 44%), %	29.44	29.44	29.44	29.44	29.44	17.09	17.09	17.09	17.09	17.09	17.43	17.43	17.43	17.43	17.43
Corn gluten meal (61%), %	3.71	3.71	3.71	3.71	3.71	6.75	6.75	6.75	6.75	6.75	5.68	5.68	5.68	5.68	5.68
Calcium bicarbonate (Anhydrous), %	1.63	1.63	1.63	1.63	1.63	1.55	1.55	1.55	1.55	1.55	1.29	1.29	1.29	1.29	1.29
Soy oil, %	4.55	4.55	4.55	4.55	4.55	1.67	1.67	1.67	1.67	1.67	3.82	3.82	3.82	3.82	3.82
Limestone, %	1.36	1.36	1.36	1.36	1.36	1.26	1.26	1.26	1.26	1.26	1.24	1.24	1.24	1.24	1.24
Salt, %	0.31	0.31	0.31	0.31	0.31	0.22	0.22	0.22	0.22	0.22	0.24	0.24	0.24	0.24	0.24
Choline chloride (50%), %	0.23	0.23	0.23	0.23	0.23	0.26	0.26	0.26	0.26	0.26	0.22	0.22	0.22	0.22	0.22
Mineral premix^a^, %	0.20	0.20	0.20	0.20	0.20	0.20	0.20	0.20	0.20	0.20	0.20	0.20	0.20	0.20	0.20
DL-Methionine, %	0.21	0.18	0.19	0.21	0.19	0.16	0.16	0.16	0.16	0.16	0.11	0.11	0.11	0.11	0.11
L-LysineHCl, %	0.25	0.25	0.25	0.25	0.25	0.39	0.39	0.39	0.39	0.39	0.30	0.30	0.30	0.30	0.30
Vitamin premix^b^, %	0.02	0.02	0.02	0.02	0.02	0.02	0.02	0.02	0.02	0.02	0.02	0.02	0.02	0.02	0.02
L-Cysteine, %	0.03	0.05	0.05	0.03	0.05	0.04	0.04	0.04	0.04	0.04	0.02	0.03	0.02	0.03	0.02
L-Phenylalanine, %	0.00	0.01	0.00	0.00	0.00	0.11	0.11	0.10	0.11	0.10	0.21	0.21	0.21	0.21	0.21
L-Threonine, %	0.08	0.08	0.07	0.08	0.08	0.14	0.14	0.14	0.14	0.14	0.12	0.12	0.11	0.12	0.11
L-Arginine, %	0.25	0.25	0.28	0.24	0.28	0.33	0.36	0.37	0.30	0.37	0.20	0.20	0.20	0.20	0.20
L-Histidine, %	0.03	0.03	0.03	0.03	0.03	0.11	0.11	0.11	0.11	0.11	0.09	0.09	0.08	0.09	0.08
L-Isoleucine, %	0.09	0.09	0.09	0.00	0.21	0.15	0.15	0.15	0.04	0.27	0.11	0.11	0.11	0.01	0.22
L-Leucine, %	0.12	0.13	0.13	0.15	0.13	0.16	0.17	0.16	0.17	0.17	0.38	0.38	0.34	0.38	0.37
L-Tryptophan, %	0.04	0.04	0.04	0.04	0.04	0.06	0.06	0.06	0.06	0.06	0.03	0.03	0.02	0.03	0.02
L-Valine, %	0.12	0.00	0.26	0.12	0.12	0.16	0.03	0.29	0.16	0.16	0.13	0.01	0.24	0.13	0.12
L-Alanine, %	0.14	0.23	0.00	0.22	0.00	0.15	0.19	0.00	0.29	0.00	0.03	0.12	0.00	0.10	0.00
Phytase (10,000 IU/g), %	0.02	0.02	0.02	0.02	0.02	0.02	0.02	0.02	0.02	0.02	0.02	0.02	0.02	0.02	0.02
Potassium carbonate, %	0.16	0.16	0.16	0.16	0.16	0.32	0.32	0.32	0.32	0.32	0.13	0.13	0.13	0.13	0.13
Sodium bicarbonate, %	0.06	0.06	0.06	0.06	0.06	0.23	0.23	0.23	0.23	0.23	0.19	0.19	0.19	0.19	0.19
Nutrient concentration (analyzed)															
SID CP, %	20.79	21.22	20.66	20.96	21.01	18.56	18.42	19.31	19.19	18.93	17.94	17.90	18.37	18.06	17.90
SID Isoleucine, %	0.81	0.83	0.76	0.68	0.85	0.70	0.83	0.71	0.68	0.87	0.73	0.71	0.77	0.64	0.81
SID Leucine, %	1.80	1.83	1.84	1.85	1.81	1.87	1.87	1.78	1.84	1.84	2.00	1.94	1.92	1.96	1.96
SID Valine, %	0.96	0.71	1.03	0.90	0.92	0.84	0.73	0.98	0.88	0.86	0.85	0.69	0.88	0.80	0.82
Nutrient concentration (calculated)															
ME, Mcal/kg	3.11	3.11	3.12	3.11	3.12	3.09	3.08	3.10	3.08	3.10	3.20	3.19	3.20	3.19	3.21
Dry matter, %	89.31	89.31	89.37	89.31	89.35	89.05	89.00	89.05	89.05	89.05	89.15	89.12	89.16	89.11	89.18
Calcium, %	1.05	1.05	1.05	1.05	1.05	0.96	0.96	0.96	0.96	0.96	0.88	0.88	0.88	0.88	0.88
Available phosphorus, %	0.35	0.35	0.35	0.35	0.35	0.33	0.33	0.33	0.33	0.33	0.28	0.28	0.28	0.28	0.28
SID Lysine, %	1.09	1.09	1.09	1.09	1.09	0.98	0.98	0.98	0.98	0.98	0.90	0.90	0.90	0.90	0.90
SID Methionine, %	0.48	0.48	0.48	0.48	0.48	0.45	0.45	0.45	0.45	0.45	0.39	0.39	0.39	0.39	0.39
SID Cysteine, %	0.28	0.31	0.27	0.27	0.27	0.29	0.29	0.29	0.29	0.29	0.26	0.26	0.26	0.26	0.26
SID Methionine + Cysteine, %	0.76	0.79	0.75	0.75	0.75	0.73	0.73	0.73	0.73	0.73	0.65	0.65	0.65	0.65	0.65
SID Phenylalanine + Tyrosine, %	1.30	1.30	1.30	1.30	1.30	1.34	1.34	1.33	1.34	1.33	1.39	1.39	1.39	1.39	1.39
SID Phenylalanine, %	0.88	0.88	0.88	0.88	0.88	0.91	0.91	0.90	0.91	0.90	0.98	0.98	0.98	0.98	0.98
SID Tyrosine, %	0.42	0.42	0.42	0.42	0.42	0.43	0.43	0.43	0.43	0.43	0.41	0.41	0.41	0.41	0.41
SID Threonine, %	0.69	0.69	0.65	0.66	0.65	0.66	0.66	0.66	0.66	0.66	0.62	0.62	0.61	0.62	0.61
SID Arginine, %	1.37	1.37	1.37	1.36	1.37	1.16	1.18	1.19	1.13	1.19	1.01	1.01	1.01	1.01	1.01
SID Histidine, %	0.49	0.49	0.49	0.49	0.49	0.50	0.50	0.50	0.50	0.50	0.47	0.47	0.47	0.47	0.47
SID Tryptophan, %	0.22	0.24	0.20	0.24	0.23	0.19	0.19	0.19	0.19	0.19	0.16	0.16	0.15	0.16	0.15
Kalium, %	0.78	0.78	0.78	0.78	0.78	0.70	0.70	0.70	0.70	0.70	0.59	0.59	0.59	0.59	0.59
Natrium, %	0.16	0.16	0.16	0.16	0.16	0.16	0.16	0.16	0.16	0.16	0.16	0.16	0.16	0.16	0.16
Chlorine, %	0.28	0.28	0.28	0.28	0.28	0.25	0.25	0.25	0.25	0.25	0.25	0.25	0.25	0.25	0.25
Choline, mg/kg	2,345.29	2,345.29	2,345.29	2,345.29	2,345.29	2,220.65	2,220.65	2,220.65	2,220.65	2,220.65	2047.55	2047.55	2047.55	2047.55	2047.55
Kalium, mmol/kg	198.69	198.69	198.69	198.69	198.69	177.80	177.80	177.80	177.80	177.80	150.98	150.98	150.98	150.98	150.98
Natrium, mmol/kg	68.73	68.73	68.73	68.73	68.73	71.26	71.26	71.26	71.26	71.26	71.21	71.21	71.21	71.21	71.21
Chlorine, mmol/kg	77.95	77.95	77.95	77.95	77.95	70.60	70.60	70.60	70.60	70.60	69.59	69.59	69.59	69.59	69.59
Electrolyte balance, mmol/kg	189.46	189.46	189.46	189.46	189.46	178.45	178.45	178.45	178.45	178.45	152.61	152.61	152.61	152.61	152.61

*LP* Low-protein control diet, *LPLV* Low-protein valine restriction diet, *LPHV* Low-protein valine supplementation diet, *LPLI* Low-protein isoleucine restriction diet, *LPHI* Low-protein isoleucine supplementation diet

^a^The Mineral premix provided the following per kg of diet: Cu: 16 mg; Zn: 110 mg; Fe: 80 mg; Mn: 120 mg; Se: 0.30 mg; I: 1.50 mg

^b^The Vitamin premix provided the following per kg of diet: Vitamin A: 15,000 IU; Vitamin D_3_: 3,600 IU; Vitamin E: 30 IU; Vitamin K_3_: 3.00 mg; Vitamin B_2_: 9.60 mg; Vitamin B_12_: 0.03 mg; Biotin: 0.15 mg; Folic acid: 1.50 mg; Pantothenic acid: 13.80 mg; Niacin: 45 mg

### Sample collection

The body weights and feed consumption of the broilers were recorded at the beginning and end of each rearing stage. At the end of the experiment, animals (1 broiler per cage) were selected for sample collection based on their average body weight. Following euthanasia by electrocution, blood was promptly collected from the jugular vein into procoagulant tubes and incubated at room temperature for 4 h before being transferred to a 4 °C centrifuge (Optima XE-90, Beckman Coulter, Inc., USA). The samples were centrifuged at 1,509 × *g* for 15 min, and the supernatant was stored at −80 °C. Subsequently, the abdominal cavity was immediately opened to collect liver and pancreas tissues, which were placed in sterile cryotubes, quenched in liquid nitrogen, and stored at −80 °C.

### Determination of serum biochemical parameters, hepatic enzyme activities, and metabolite contents

Serum concentrations of BCAAs were measured using an automatic amino acid analyzer (Hitachi L-8800, Hitachi, Japan), and all procedures were performed according to the manufacturer's instructions. Commercial enzyme-linked immunosorbent assay kits (Nanjing Jiancheng Bioengineering Institute, Nanjing, China) were used to determine: 1) serum levels of insulin, insulin-like growth factor 1, interleukin (IL)-6, glucose, uric acid, oxypurinol, and pantothenic acid; 2) enzymatic activities of BCAT, BCKDH, aspartate aminotransferase, alanine aminotransferase, alkaline phosphatase, and gamma-glutamyl transpeptidase in the liver; 3) contents of fatty acids, high-density lipoprotein, low-density lipoprotein, total cholesterol, triglycerides, acylcarnitine, resistin, plasminogen activator inhibitor-1, insulin receptor 1, IL-1β, IL-6, and tumor necrosis factor-alpha (TNF-α) in the liver; and 4) contents of IL-6, insulin, glucagon, and glucagon-like peptide-1 (GLP-1) in the pancreas. All procedures were performed strictly according to the manufacturer's protocol.

### Hepatic histological examination

Liver tissues were harvested from broilers in the designated treatment groups, immediately fixed in 4% paraformaldehyde, embedded in paraffin, sectioned, and stained with hematoxylin-eosin and Oil Red O. The liver sections were imaged using an EVOS M7000 microscope (Thermo Fisher Scientific, Waltham, MA, USA).

### Real-time fluorescence quantification

RNA extraction, reverse transcription, and real-time fluorescence quantitative PCR were performed as previously described by Yang et al. [17]. Total RNA was extracted from the liver and pancreatic tissues using RNAiso Plus reagent (Takara). RNA purity and concentration were determined using a nucleic acid analyzer, followed by cDNA synthesis using a reverse transcription kit. The cDNA was diluted for qRT-PCR analysis. mRNA expression levels were normalized to those of glyceraldehyde-3-phosphate dehydrogenase, and the relative expression levels of the remaining mRNAs were calculated. Primers were designed based on sequences retrieved from GenBank and were synthesized by Sangon Biotechnology (Shanghai, China). The specific information is presented in Table 2.

**Table 2:** Parameters of primer pairs for RT-PCR

Genes^a^	Genbank number	Sequence (5'→3')
*GAPDH*	NM_204305.1	F: GAGGGTAGTGAAGGCTGCTG
		R: CATCAAAGGTGGAGGAATGG
*LAMB2*	NM_002292.4	F: TCCCTGTTCCCCTCTTCAGG
		R: GGCAGCCAGCACGCTTA
*ITGA*	NM_001033228.3	F: CGCTGTGAATCAGACGAGGT
		R: CCCACAGGGCTCATTCTTGT
*PI3K*	XM_028674370	F: CTTCTGGAGTCCTATTGTCG
		R: CACCTTCTGGGTCTCATCTT
*AKT*	NM_001300425.1	F: GCCGTGAGCCCAGTTAGG
		R: AGCTACTTATGGCTGCGGGA
*mTOR*	NM_004958.4	F: GTGGCGATCCTATGGCATGA
		R: ACGCCTGAAAACGTGGTAGT
*LPIN*	NM_001201953.3	F: CCCACGATCTGGAAGTCTGG
		R: TCTGAGATACGGCAACTGCT
*DGAT*	XM_028676226.1	F: AGCTGAAGTTACGAGATTTGGAGA
		R: ACTCAGCAAACGAACTGGCA
*PP2A*	NM_105665.3	F: CGGTGAGAACATGGACCAGA
		R: GAGTGGTTTCGGGTTCGACT
*NF-κB*	NM_001410442.1	F: GGGATGCGGTTCCGCTATAA
		R: TCACCAAAGAGACCCGAACG
*CCL20*	NM_004591.3	F: GCGAATCAGAAGCAGCAAGC
		R: GATGTCACAGCCTTCATTGGC
*FZD*	NM_080073.3	F: GCTGCTTGTTTACGGTGCTC
		R: CCACGTAGGCACATCCAACT
*Axin*	NM_001078192.1	F: CAGAGGATCGCACAGGTCTC
		R: GGCATCATGTCCTGGGCTAA
*Gcg*	NM_002054.5	F: GTTCAAGGCAGCTGGCAAAATCCT
		R: TCCTCGTCCATTCACTAACCAAGC
*PC2*	NM_001204470.1	F: CTCACCTCCAAAAGGAACCA
		R: CCCACCTTGGAATCATCATC
*PC1/3*	NM_000439.5	F: ATGGCTTGGAGTGGAATCAC
		R: CAAAAATGGAGCCTTTTCCA
*GLP-1R*	NM_002062.5	F: GCTGCTGGAGCAGGAACTAT
		R: TGTTGGCTGGACACTTCAGA
*PKA*	XM_002286119.1	F: CGTTCACTCTCTGTGGCACT
		R: CAACTCCCTTGCCATGTCCT

^a^*GAPDH* Glyceraldehyde-3-phosphate dehydrogenase, *LAMB2* Laminin beta2, *ITGA* Integrin alpha, *PI3K* Phosphatidylinositol 3-kinase, *AKT* Serine/threonine-protein kinase, *mTOR* Mechanistic target of rapamycin kinase, *LPIN* Phosphatidate phosphatase, *DGAT* Diacylglycerol O-acyltransferase, *PP2A* Serine/threonine protein phosphatase 2 A, *NF-κB* Nuclear factor of kappa light polypeptide gene enhancer in B cells, *CCL20* C–C motif chemokine ligand 20, *FZD* Frizzled class receptor, *Gcg* Glucagon, *PC2* Prohormone convertase 2, *PC1/3* Prohormone convertase 1/3, *GLP-1R* Glucagon like peptide 1 receptor, *PKA* cAMP dependent protein kinase

### Transcriptomic analysis

#### Sample extraction and sequencing

Total RNA was enriched for poly (A)-containing mRNA using oligo (dT)-conjugated magnetic beads. RNA was fragmented to approximately 300 bp using an ionization-based method, and 300 bp fragments were selected. The first strand of cDNA was synthesized using a 6-base random primer and reverse transcriptase with RNA as the template, followed by synthesis of the second cDNA strand using the first strand as the template. After library construction, PCR amplification was performed to enrich the library fragments, and size selection was performed to obtain libraries with a fragment size of 450 bp. Library quality was assessed using an Agilent 2100 Bioanalyzer, and the total and effective library concentrations were measured. Based on the effective concentration and required data volume, libraries with unique index sequences (each sample was assigned a distinct index to differentiate the downstream data) were pooled. Pooled libraries were uniformly diluted to 2 nmol/L and converted into single-stranded libraries via alkaline denaturation. Following RNA extraction, purification, and library preparation, sequencing was performed using second-generation sequencing technology (Next-Generation Sequencing, NGS) on an Illumina platform with paired-end (PE) sequencing.

#### Transcriptomic data analysis

Raw sequencing data (Raw Data) were filtered by ProteoWizard software to remove low-quality reads, and high-quality sequences (Clean Data) were aligned to the reference genome of the species. Gene expression levels were quantified based on alignment results. Differential expression, enrichment, and clustering analyses were conducted by Variable Importance in the Projection (VIP) value of OPLS-DA model (VIP ≥ 1) and independent sample *t*-test (*P* < 0.05). Successfully aligned reads were assembled to reconstruct the transcript sequences.

### Lipidomics analysis

#### Sample pretreatment

A 20 mg liver sample was taken, and 200 μL of water was added. The mixture was vortexed using an MP homogenizer, followed by the addition of 800 μL of MTBE. After vortexing and mixing, 240 μL of pre-cooled methanol was added, and the solution was vortexed again. Samples were sonicated in a 4 °C water bath for 20 min and incubated at room temperature for 30 min. Subsequently, the sample was centrifuged at 14,000 × *g* at 10 °C for 15 min. The upper organic phase was collected and dried under a nitrogen atmosphere. Before mass spectrometry analysis, the dried sample was reconstituted with 200 μL of a 90% isopropanol/acetonitrile solution. The reconstituted solution was thoroughly vortexed, and 90 μL was taken for further processing. The solution was centrifuged again at 14,000 × *g* at 10 °C for 15 min, and the supernatant was collected for injection analysis.

#### Injection conditions

The samples were separated using the Nexera LC-30A ultra-high performance liquid chromatography system equipped with a C18 column. The column temperature was maintained at 45 °C, and the flow rate was set to 300 μL/min. The mobile phase consisted of two components: A (acetonitrile–water solution, 6:4 v/v + 0.1% formic acid + 0.1 mmol/L ammonium formate) and B (acetonitrile-isopropanol solution, 1:9 v/v + 0.1% formic acid + 0.1 mmol/L ammonium formate). Detection was performed in both positive and negative ion modes using electrospray ionization. After separation mass spectrometry analysis was performed using a Q Exactive series mass spectrometer (Thermo Scientific™). The mass-to-charge ratios of lipid molecules and lipid fragments were acquired as follows: ten fragment spectra (MS2 scan, HCD) were collected after each full scan. The resolution of MS1 was set to 70,000 at *m/z* 200, and that of MS2 was set to 17,500 at *m/z* 200.

#### Lipidomics data analysis

The LipidSearch software was used for peak identification, peak extraction, and lipid identification (secondary identification) of lipid molecules and internal standard lipid molecules. The main parameters were as follows: precursor tolerance, 5 mg/kg; product tolerance, 5 mg/kg; and product ion threshold, 5%. The data extracted using LipidSearch were subjected to quality evaluation and further analysis.

### Statistical analysis

Each cage was considered an experimental unit. All experimental data are expressed as means. Data analysis was performed using one-way ANOVA using the SPSS software (version 27.0). *P* < 0.05 was considered to indicate statistical significance, while 0.05 < *P* < 0.1 was considered to indicate a trend toward significance. The analyzed data were visualized as figures using GraphPad Prism 8.

## Results

### Isoleucine and valine exhibited specific roles in liver metabolism and insulin function

In this study, significant differences in growth performance were observed among 5 treatments across all growth stages (*P* < 0.05) (Table 3). Compared with the LP, LPHV and LPLI exhibited marked improvements in growth performance, as evidenced by significantly higher final body weights (*P* < 0.05) and lower feed conversion ratios (*P* < 0.05). These results suggest that modulating diet Val and Ile concentrations could effectively mitigate the antagonistic of BCAA.

**Table 3:** Effects of different valine/isoleucine allowance on growth performance of AA broilers

Stage	Item	LP	LPLV	LPHV	LPLI	LPHI	SEM	*P*-value
0–14 d	IBW, kg	0.047	0.047	0.047	0.047	0.047	0.000	0.425
	FBW, kg	0.502^ab^	0.479^b^	0.485^b^	0.522^a^	0.499^ab^	0.004	0.039
	ADFI, kg	0.038^b^	0.038^b^	0.038^b^	0.041^a^	0.040^ab^	0.000	0.060
	ADG, kg	0.032^ab^	0.031^b^	0.030^b^	0.034^a^	0.032^ab^	0.000	0.081
	FCR	1.196	1.226	1.249	1.212	1.246	0.007	0.156
15–28 d	FBW, kg	1.517^ab^	1.442^b^	1.556^a^	1.580^a^	1.504^ab^	0.014	0.011
	ADFI, kg	0.111^bc^	0.117^ab^	0.123^a^	0.118^ab^	0.116^ab^	0.001	0.036
	ADG, kg	0.065^b^	0.068^b^	0.077^a^	0.077^a^	0.070^b^	0.001	0.002
	FCR	1.717^a^	1.714^a^	1.595^b^	1.537^b^	1.650^ab^	0.022	0.007
29–42 d	FBW, kg	2.867^ab^	2.731^c^	2.939^ab^	2.961^ab^	2.851^b^	0.024	0.005
	ADFI, kg	0.177^c^	0.185^ab^	0.194^a^	0.188^ab^	0.184^ab^	0.002	0.023
	ADG, kg	0.082^b^	0.086^b^	0.097^a^	0.098^a^	0.089^b^	0.002	0.003
	FCR	2.214^a^	2.214^a^	2.056^bc^	1.987^bc^	2.133^ab^	0.029	0.007
0–42 d	ADFI, kg	0.109^bc^	0.113^ab^	0.118^a^	0.116^ab^	0.113^ab^	0.001	0.017
	ADG, kg	0.060^c^	0.062^c^	0.068^ab^	0.070^a^	0.064^bc^	0.001	0.002
	FCR	1.824^a^	1.835^a^	1.737^ab^	1.668^b^	1.778^a^	0.020	0.005

*LP* Low-protein control diet, *LPLV* Low-protein valine restriction diet, *LPHV* Low-protein valine supplementation diet, *LPLI* Low-protein isoleucine restriction diet, *LPHI* Low-protein isoleucine supplementation diet, *IBW* Initial body weight, *FBW* Final body weight, *ADFI* Average daily feed intake, *ADG* Average daily gain, *FCR* Feed:Gain

^a–c^Dissimilar letters represent significant difference among different treatments (*P* < 0.05)

**Fig. 1:**
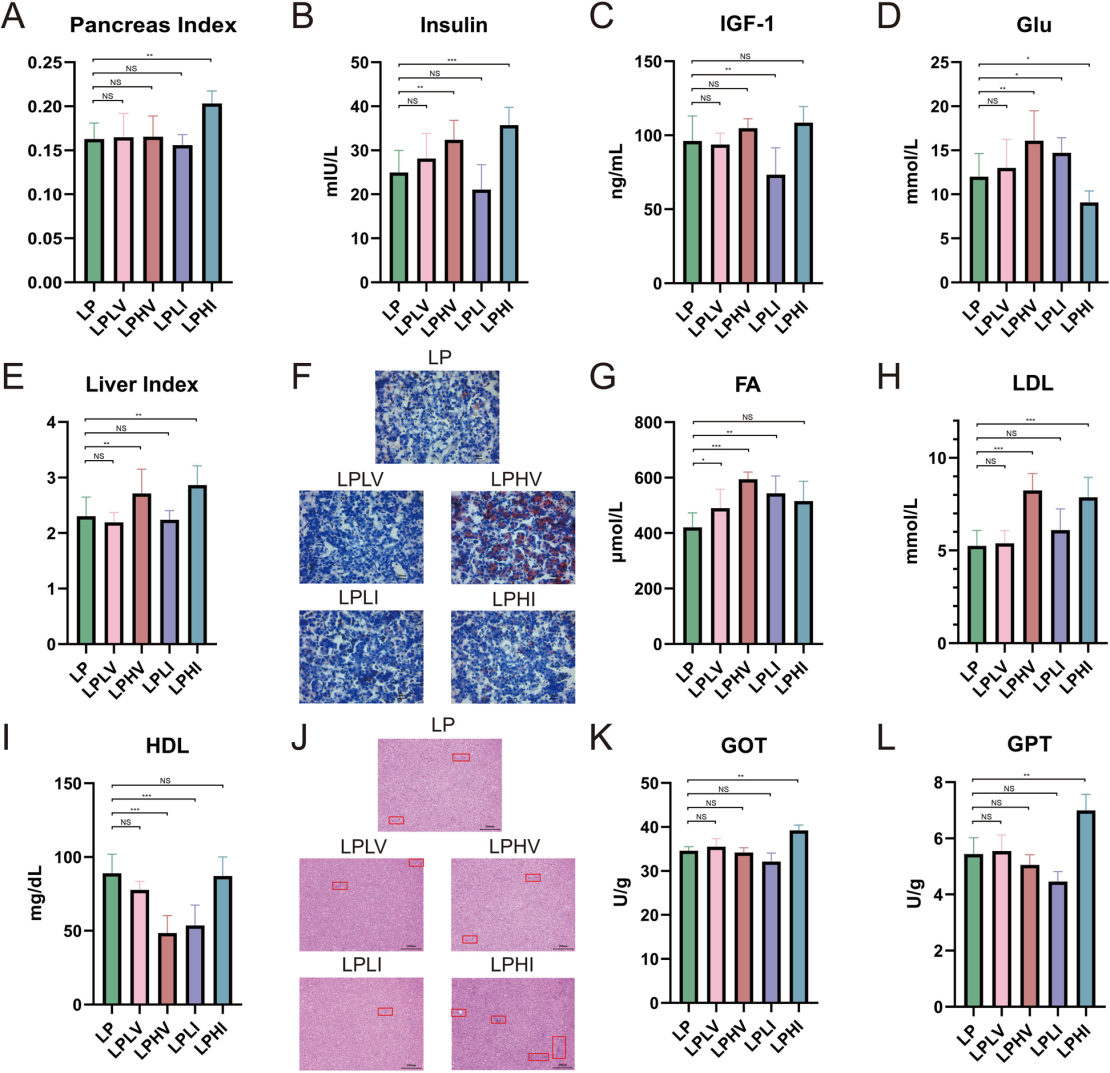
Isoleucine and valine alter hepatic metabolism and insulin function. **A** Pancreas index. **B** Serum insulin concentration. **C** Serum insulin-like growth factor 1 concentration. **D** Serum glucose concentration. **E** Liver index. **F** Hepatic tissue section stained with Oil Red. **G** Hepatic fatty acid concentration. **H** Hepatic low-density lipoprotein concentration. **I** Hepatic high-density lipoprotein concentration. **J** Hepatic tissue section stained with hematoxylin–eosin. **K** Hepatic glutamic oxalacetic transaminase activity. **L** Hepatic glutamic-pyruvic transaminase activity. LP: Low-protein control diet; LPLV: Low-protein valine restriction diet; LPHV: Low-protein valine supplementation diet; LPLI: Low-protein isoleucine restriction diet; LPHI: Low-protein isoleucine supplementation diet. An asterisk (*) denotes *P* < 0.1; double asterisks (**) denote* P* < 0.05; triple asterisks (***) denote *P* < 0.01; and "NS" denotes no significant difference

After Ile supplementation, the pancreatic index significantly increased (*P* < 0.05; Fig. 1A), leading to an expected increase in serum insulin and insulin-like growth factor 1 levels (*P* < 0.05; Fig. 1B and C) and a decrease in blood glucose levels (*P* < 0.05; Fig. 1D). This indicates that Ile may promote pancreatic development and insulin secretion without negatively affecting insulin function. In contrast, Val had a minimal impact on the pancreatic index. Moreover, despite elevated serum insulin levels after Val supplementation, the corresponding blood glucose levels did not decrease as anticipated (*P* < 0.05; Fig. 1D), suggesting that Val may potentially inhibit normal insulin function. The increased insulin content observed in the high-Val group may be attributed to compensatory secretion due to impaired insulin function. Surprisingly, the liver index increased significantly after supplementation with both Ile and Val (*P* < 0.05; Fig. 1E), indicating that both BCAAs exert negative effects on the liver. To explore their specific impacts on liver metabolism, we performed Oil Red O staining of liver tissues. Red lipid droplets in the liver markedly increased after Val supplementation and decreased after Val restriction (Fig. 1F). Correspondingly, FA and low-density lipoprotein contents were significantly elevated and high-density lipoprotein was depressed after Val supplementation (*P* < 0.05; Fig. 1G–I), whereas no significant changes were observed with Ile adjustment. These findings suggest that Val may induce adverse reactions via hepatic fat accumulation, whereas Ile appears to have little effect on fat metabolism. Subsequently, we conducted hematoxylin–eosin staining of liver tissues to investigate the specific effects of Ile. As Ile levels increased, inflammatory cells progressively accumulated in the liver, accompanied by evident inflammatory cell infiltration and vacuolation (Fig. 1J). Consistently, the activities of serum glutamic-oxaloacetic transaminase and glutamic-pyruvic transaminase were significantly increased (*P* < 0.05; Fig. 1K and L), indicating that Ile may cause adverse effects by promoting liver inflammation.

### The protein kinase B (AKT)/mTOR signaling pathway was the common route for Val and Ile to induce liver metabolic changes

**Fig. 2:**
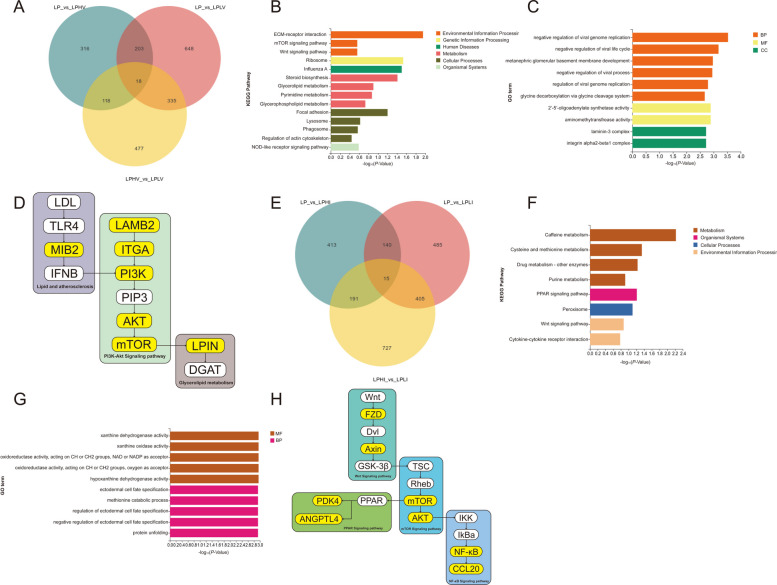
Valine and isoleucine supplementation reprograms hepatic metabolism. **A** Venn plot of LP vs. LPLV and LP vs. LPHV shared liver significantly altered genes. **B** Kyoto Encyclopedia of Genes and Genomes enrichment analysis of shared liver significantly altered genes in LP, LPLV, and LPHV. **C** Gene Ontology enrichment analysis of shared liver significantly altered genes in LP, LPLV, and LPHV. **D** Pathways map of significantly altered genes in LP, LPLV, and LPHV. **E** Venn plot of LP vs. LPLI and LP vs. LPHI shared liver significantly altered genes. **F** Kyoto Encyclopedia of Genes and Genomes enrichment analysis of shared liver significantly altered genes in LP, LPLI, and LPHI. **G** Gene Ontology enrichment analysis of shared liver significantly altered genes in LP, LPLI, and LPHI. **H** Pathways map of significantly altered genes in LP, LPLI, and LPHI. LP: Low-protein control diet; LPLV: Low-protein valine restriction diet; LPHV: Low-protein valine supplementation diet; LPLI: Low-protein isoleucine restriction diet; LPHI: Low-protein isoleucine supplementation diet

Based on the preliminary understanding that excessive BCAA exerted negative effects on the liver, we conducted transcriptomic analyses of liver tissues after individually adjusting for dietary Val and Ile levels to further elucidate the specific mechanisms underlying liver metabolic changes. To highlight the dose-dependent changes induced by BCAA, we identified 18 and 15 common differentially expressed genes (DEGs) among the three treatment groups for Val (LP, LPLV, and LPHV) and Ile (LP, LPLI, and LPHI), respectively (Fig. 2A and E). Subsequently, Kyoto Encyclopedia of Genes and Genomes and Gene Ontology enrichment analyses were performed on these DEGs to explore their potential molecular functions and pathways (Fig. 2B, C, F, and G). In the Val-adjusted groups, the molecular functions of the DEGs were primarily enriched in biological processes related to oligoadenylate synthetase and aminomethyltransferase activities, with metabolic pathways mainly involving glycerolipid metabolism, glycerophospholipid metabolism, and the mTOR signaling pathway (Fig. 2B and C). Similarly, in the Ile-adjusted groups, the molecular functions of the DEGs were predominantly enriched in purine metabolism-related biological processes, with metabolic pathways primarily involving the Wnt, PPAR, and cytokine signaling pathways (Fig. 2F and G). These results revealed detailed transcriptional-level alterations in liver metabolism caused by Val and Ile, consistent with previous findings that Val promotes liver fat accumulation and Ile induces liver inflammation. After excluding unrelated genes, we constructed gene pathway diagrams under the influence of Val and Ile (Fig. 2D and H). From these diagrams, it was evident that, although the DEGs regulated by Val and Ile were distributed across different pathways, they both clearly converged in the AKT/mTOR signaling pathway. The AKT signaling pathway is closely associated with insulin signaling, whereas the mTOR signaling pathway has been extensively demonstrated in numerous studies to be intricately linked to BCAA metabolism. This finding underscores that despite the distinct metabolic directions of Val and Ile, their effects are still centered on the established functions of BCAAs, providing robust evidence for our earlier hypothesis.

### Val promoted ceramide accumulation and inhibited insulin signaling

**Fig. 3:**
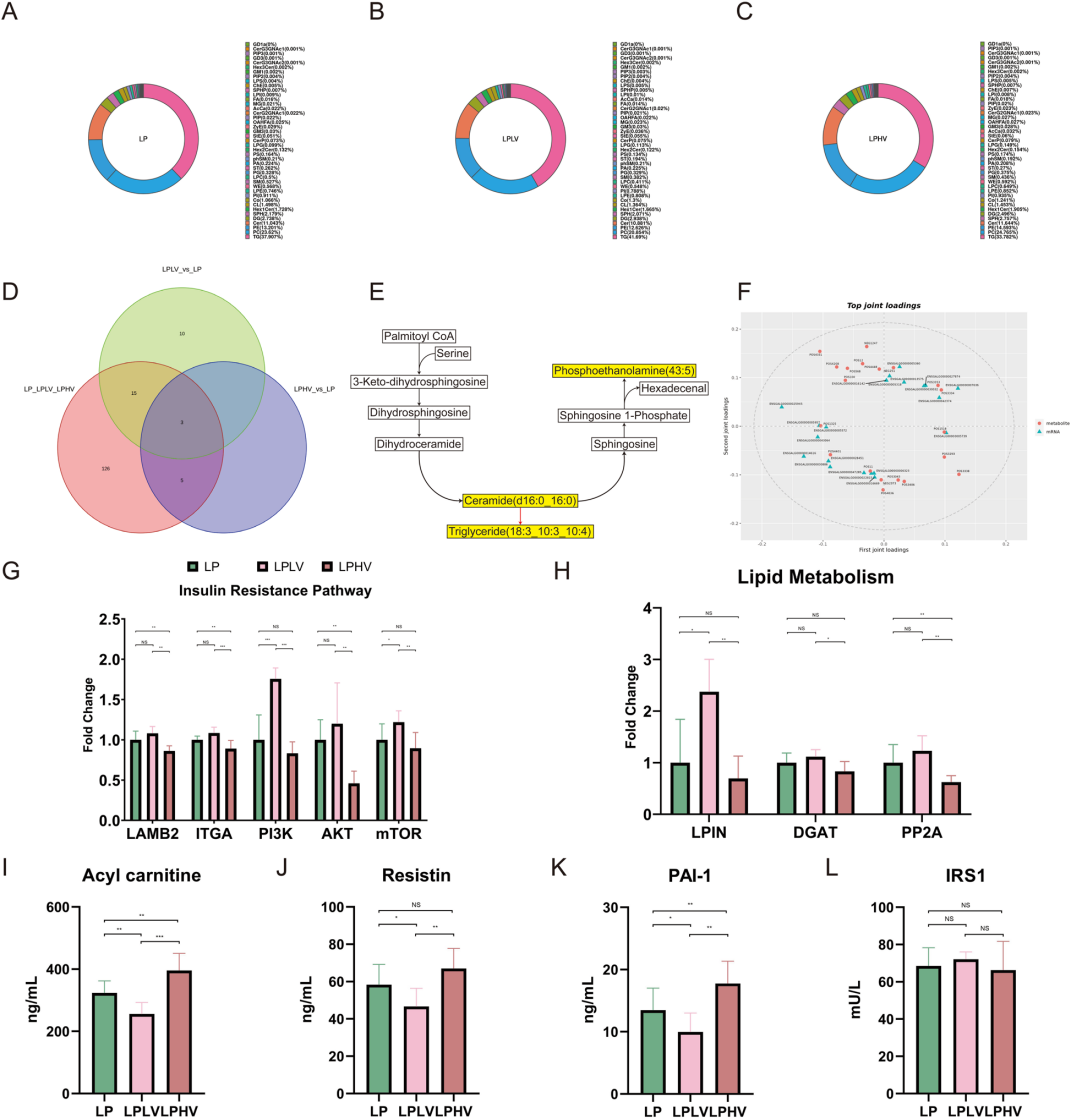
Valine supplementation regulates lipid metabolism and causes hepatic insulin resistance. **A** Lipid composition analysis of LP liver lipid metabolome data. **B** Lipid composition analysis of LPLV liver lipid metabolome data. **C** Lipid composition analysis of LPHV liver lipid metabolome data. **D** Venn plot of LP vs. LPLV and LP vs. LPHV shared liver significantly altered metabolites. **E** Significantly altered metabolites mechanism map. **F** Top 20 loading elements of transcriptome and metabolome linkage analysis. **G** Hepatic insulin resistance pathway-related mRNA expression. **H** Hepatic lipid metabolism-related mRNA expression. **I** Hepatic acyl carnitine concentration. **J** Hepatic resistin concentration. **K** Hepatic plasminogen activator inhibitor-1 concentration. **L** Hepatic insulin receptor substrate 1 concentration. LP: Low-protein control diet; LPLV: Low-protein valine restriction diet; LPHV: Low-protein valine supplementation diet. An asterisk (*) denotes *P* < 0.1; double asterisks (**) denote* P* < 0.05; triple asterisks (***) denote *P* < 0.01; and "NS" denotes no significant difference

Non-targeted lipidomics serves as an excellent tool for exploration and provides detailed insights into changes in lipid molecules within the liver. Lipid subclass analysis revealed significant differences in the four components of hepatic fat, triglycerides (TG), phosphatidylcholine (PC), phosphatidylethanolamine (PE), and ceramide (Cer), after Val adjustment. The TG content decreased significantly following Val supplementation (Fig. 3A–C), suggesting that Val may regulate lipid metabolism by altering the direction of lipid synthesis. Subsequently, we identified three common differential lipid metabolites across the groups: Cer(d16:0_16:0), PE(43:5), and TG(18:3_10:3_10:4) (Fig. 3D). Based on these metabolites, a metabolic mechanism diagram illustrating the transformation and synthesis pathways was constructed (Fig. 3E). Palmitoyl-CoA, a precursor for lipid molecule synthesis, is redirected toward ceramide production upon Val supplementation, inhibiting triglyceride generation, and ultimately increasing phosphatidylethanolamine levels. Based on this, we performed an integrated lipidomics and transcriptomics analysis, selecting genes associated with differential lipid metabolites based on the load values (Fig. 3F). This approach provides robust evidence that links lipidomic and transcriptomic changes to an overall biological process. The qPCR validation confirmed the reliability of the transcriptomic analysis, and the results were consistent with those of previous studies. Specifically, Val supplementation disrupted the PI3K/AKT axis in insulin signaling and significantly altered the expression levels of transcription factors involved in fat metabolism (*P* < 0.05; Fig. 3G and H). Additionally, the upstream regulators LAMB2 and ITGA exhibited significantly reduced expression, indicating that Val may suppress PI3K/AKT signaling through both direct effects and alterations in lipid metabolism. Impaired insulin signaling is closely associated with insulin resistance. Acylcarnitine, resistin, and plasminogen activator inhibitor-1 are sensitive markers for assessing insulin resistance. We observed increased hepatic levels of these three substances after Val supplementation (*P* < 0.05; Fig. 3I, J, and K), confirming the occurrence of insulin resistance under these conditions. Furthermore, insulin receptor analysis revealed no changes in receptor function, suggesting that the observed resistance was not receptor-mediated (*P* > 0.05; Fig. 3L). In summary, Val induces insulin resistance through specific accumulation of hepatic lipids, thereby inhibiting insulin signaling pathways.

### Ile promoted insulin secretion via IL-6 release

**Fig. 4:**
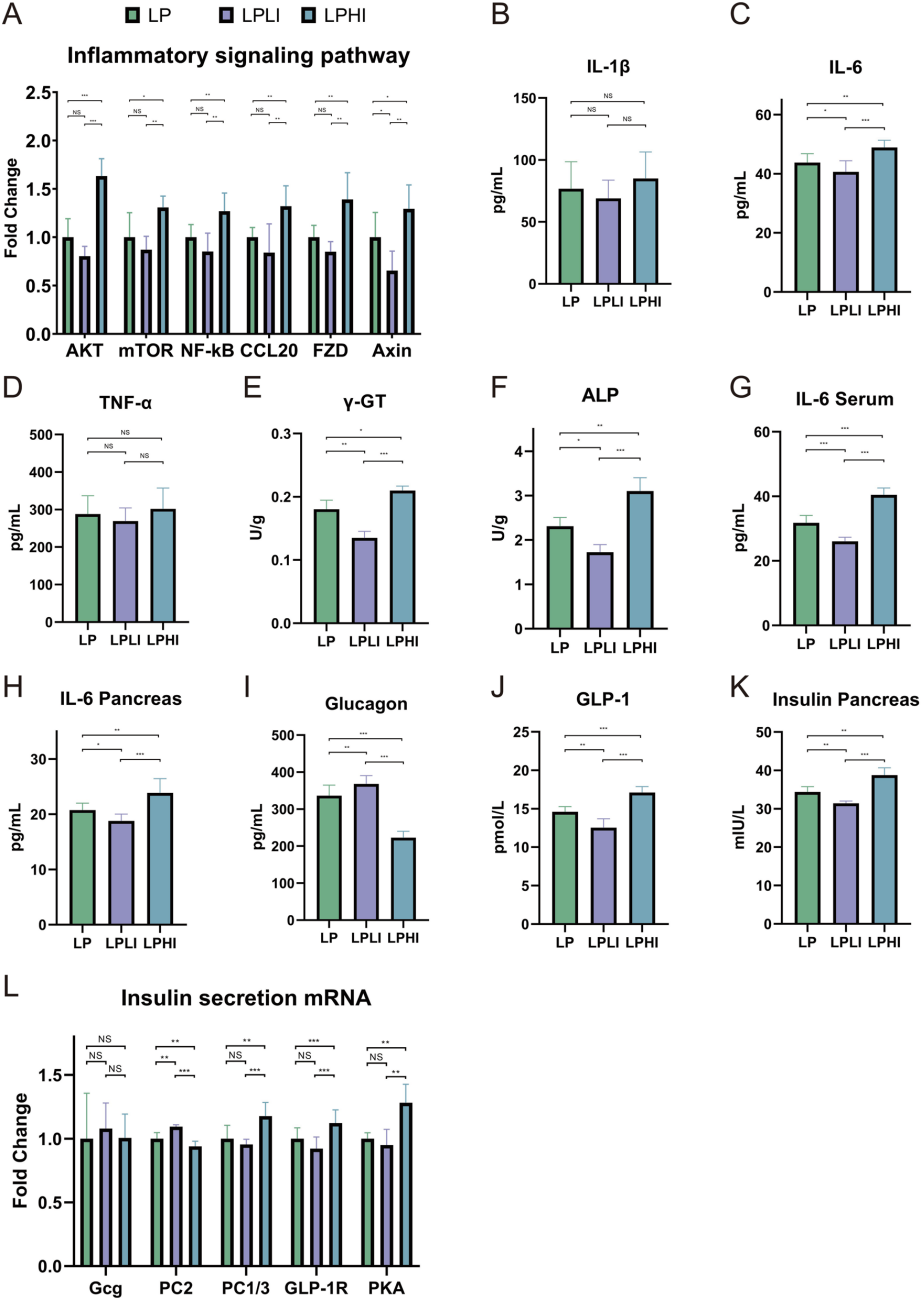
Isoleucine supplementation promotes insulin secretion. **A** Hepatic inflammatory signaling pathway-related mRNA expression. **B** Hepatic interleukin-1 beta concentration. **C** Hepatic interleukin-6 concentration. **D** Hepatic tumor necrosis factor-alpha concentration. **E** Hepatic gamma-glutamyl transpeptidase activity. **F** Hepatic alkaline phosphatase activity. **G** Serum interleukin-6 concentration. **H** Pancreatic interleukin-6 concentration. **I** Pancreatic glucagon concentration. **J** Pancreatic glucagon-like peptide-1 concentration. **K** Pancreatic insulin concentration. **L** Pancreatic insulin secretion related mRNA expression. LP: Low-protein control diet; LPLI: Low-protein isoleucine restriction diet; LPHI: Low-protein isoleucine supplementation diet. An asterisk (*) denotes *P* < 0.1; double asterisks (**) denote* P* < 0.05; triple asterisks (***) denote *P* < 0.01; and "NS" denotes no significant difference

After elucidating the mechanism by which Ile regulates hepatic transcriptional changes, we investigated the specific mechanisms underlying the promotion of insulin secretion. Initially, we validated these findings using qPCR (*P* < 0.05; Fig. 4A), confirming that Ile supplementation significantly upregulated AKT/mTOR expression and activated the downstream nuclear factor-kappa B (NF-κB) inflammatory signaling pathway. Subsequently, we examined the phenotypes of the inflammatory responses and associated metabolites. Changes in gamma-glutamyl transpeptidase and alkaline phosphatase activities reaffirmed the occurrence of liver inflammation (*P* < 0.05; Fig. 4E and F). Among the three inflammatory cytokines (IL-1β, IL-6, and TNF-α), only IL-6 levels were altered (*P* < 0.05; Fig. 4B–D), emphasizing the specificity of IL-6 release in Ile-induced pro-inflammatory effects. Given the reports linking inflammatory factors to insulin secretion, we explored whether similar changes occurred in the pancreas, focusing on IL-6. The observed changes in IL-6 levels in both the blood and pancreatic tissues supported our hypothesis (*P* < 0.05; Fig. 4G and H), indicating that IL-6 was released from the liver and transported to the pancreas via the bloodstream. The pancreas primarily produces two hormones, insulin and glucagon, which are synthesized from proglucagon (Gcg) through distinct pathways and exhibit antagonistic interactions. Thus, analyzing the tissue-level changes in these hormones provides critical insights into the mechanisms of insulin secretion. Our results demonstrated a significant shift in the balance between glucagon, insulin, and the secretory precursor GLP-1 (*P* < 0.05; Fig. 4I–K). mRNA expression analysis (Fig. 4L) confirmed that IL-6 promoted insulin secretion by inhibiting *PC2* expression and glucagon production without altering Gcg levels, thereby enhancing the conversion of proglucagon to GLP-1.

## Discussion

Our research demonstrates that adjusting the levels of different BCAAs in low-protein diets could alleviate BCAA antagonism. This suggests that there are significant functional and metabolic differences among different branched-chain amino acids, and the existence of an intermediate factor of BCAAs and their catabolic enzymes that differentiate the catabolic potential of the three amino acids. Traditionally, insulin has been regarded as a proanabolic hormone for three major macronutrients: carbohydrates, proteins, and fats [18]. However, emerging evidence indicates that insulin promotes the oxidation and catabolism of BCAAs [19]. Shin further confirmed the critical role of insulin in enhancing the activity of BCAA-catabolic enzymes [10]. These findings underscore the scientific rationale for selecting insulin as an intermediary for investigating BCAA antagonism.

Previous studies have suggested that Val supplementation had a greater alleviation effect on antagonism than Ile; however, in this study, we demonstrated that restricting Ile was an effective strategy to resolve the antagonism. These findings highlight the opposing roles of Ile and Val in insulin function. Most studies on BCAAs and insulin function have primarily focused on Leu, whereas the significant differential contributions of Ile and Val have been largely overlooked. Ile is an isomer of Leu that exhibits similar functions in various aspects [20]. Doi et al. have reported that treating C2C12 myotubes with Ile significantly increased the GLUT4 content and enhanced the glucose uptake ability [21]. Additionally, oral administration of Ile markedly reduced blood glucose levels, with better efficacy observed when combined with insulin [22]. These findings confirm that Ile has effects comparable to those of Leu in terms of improving insulin function. In this study, we verified the beneficial effects of Ile in promoting insulin function and found that dietary Ile supplementation could increase serum insulin content, suggesting that Ile may play a role similar to that of Leu in promoting insulin secretion. Conversely, the beneficial effects of Val on insulin function have rarely been reported, whereas its detrimental effects on insulin function have been extensively documented [15]. Feeding rats with Val resulted in a significant increase in blood glucose concentration without altering insulin levels [23], and Val treatment did not affect the glucose uptake ability in rats [24]. Consistent with these findings, we observed that Val likely induced insulin resistance.

BCAAs, particularly Leu, are closely associated with the activation of mTOR signaling [25]. The mTOR promotes protein synthesis and regulates cell growth, proliferation, and survival. However, it is also associated with oxidative stress and release of inflammatory factors [26]. In previous studies, the relationship between Ile and Val and mTOR has received limited attention. In this study, changes at the transcriptional level caused by excessive levels of Ile and Val in the liver of broiler chickens were observed using liver transcriptome analysis.

Val disrupts insulin signaling by inhibiting the PI3K/AKT/mTOR pathway. The PI3K/mTOR pathway plays a critical role in cell function and glucose metabolism and serves as a major insulin signaling mediator [27]. This pathway includes LAMB2 and ITGA, which are the key components of cellular processes. LAMB2 is a subunit of laminin, an extracellular glycoprotein that maintains normal cell structure and barrier integrity [28], whereas ITGA is a subunit of the integrin heterodimeric transmembrane protein complex that mediates interactions between cells and the extracellular matrix [29]. Val may disrupt PI3K signaling by interfering with normal intercellular functions. However, given that LAMB2 is predominantly expressed in the kidneys and has limited effects on the liver [28], and that ITGA primarily functions in muscle cells, it is insufficient to attribute the disruption of PI3K signaling solely to the inhibition of these two transcription factors. LPIN, a transcriptional co-regulator of phosphatidic acid phosphatase [30], exhibited significantly higher expression levels, warranting further investigation. Lipidomic analysis revealed that Val induces insulin resistance by regulating lipid metabolism, specifically by disrupting insulin signaling pathways. TG, the primary end products of lipid metabolism, constitute a substantial proportion of lipids [31]. Under the influence of Val, the TG content decreased, highlighting the role of Val in altering lipid synthesis. TG synthesis is driven by substrates such as fatty acids [32], and the intermediate 3-hydroxyisobutyrate produced during Val catabolism effectively enhances fatty acid uptake in the liver and muscle [33]. This could explain the reduction in TG synthesis observed with high-Val supplementation. Although the relationship between TG and insulin resistance has been gradually decoupled [34], and other lipid metabolites may contribute to insulin resistance, our results indicate that Val promotes Cer accumulation. Ceramide, a core component of sphingolipid synthesis and a regulator of cellular stress [35], has been shown to hinder insulin signaling and inhibit glucose uptake and storage [36]. Furthermore, extensive cell culture studies have demonstrated that ceramide antagonizes insulin action by inhibiting AKT signaling through two independent mechanisms: 1) activating protein phosphatase 2 A to dephosphorylate AKT and 2) blocking AKT translocation to the plasma membrane [37].

It has been reported that Ile activates mTOR and the downstream NF-κB inflammatory pathway via Wnt signaling, thereby inducing liver inflammation. FZD and Axin are key components of the Wnt signaling pathway. FZD proteins consist of seven transmembrane receptors, with a cysteine-rich N-terminal extracellular domain serving as an interaction site for Wnt ligands [38]. Axin primarily functions as a scaffold protein containing the binding domains of the β-catenin destruction complex [39]. Their significant expression suggests that Ile activates and propagates the Wnt pathway by promoting interactions between transcription factors within this pathway. Although BCAA is widely recognized as being directly linked to mTOR activation, our results indicate that the promoting effect of Ile is more concentrated in the upstream Wnt signaling pathway. This may explain why despite sharing many common functions with Leu, Ile exhibits a relatively weaker effect. NF-κB is a canonical pro-inflammatory signaling pathway that regulates the release of interleukins and tumor necrosis factors [40]. CCL20, a chemokine mediated by the lymphotoxin β receptor, depends on IKKα-mediated activation of the RelB/p52 complex [41]. The pro-inflammatory effect of Ile does not occur through classical NF-κB signaling but rather via an alternative mechanism involving IKKα-mediated activation of the RelB/p52 dimer [42]. This may explain why only IL-6 is released among the three typical inflammatory cytokines (IL-1β, IL-6, and TNF-α). IL-6, a pleiotropic cytokine frequently involved in immune regulation [43], plays a critical role in glucose metabolism. Recent studies have indicated that blood IL-6 levels can be used to predict the onset of type 2 diabetes [44], a characteristic highly similar to that of BCAA, suggesting a potential underlying mechanism linking IL-6 and insulin function. Glucagon and insulin are secreted by pancreatic α and β cells, respectively, and exert opposing effects on glucose homeostasis. Pancreatic α cells are primary targets of IL-6, and IL-6 downregulates zinc transporter SLC39A5 by activating STAT3, reducing cellular zinc concentration and potassium channel activity, and ultimately promoting glucagon secretion [43]. However, our findings indicate that IL-6 enhances GLP-1 release by promoting the expression of the prohormone convertase PC1/3, thereby stimulating insulin secretion. The discrepancy between research results is closely related to the source of IL-6 and the pathological state of the subjects. For instance, IL-6 improves insulin resistance in diabetic mouse models [45] but promotes blood glucose elevation in exercise-induced mouse models [46].

## Conclusion

In summary, Ile and Val modulated antagonism through influencing the insulin signaling pathway. This finding not only offers a strategy to ensure adequate amino acid nutrition in low-protein diets but also highlights the complex interplay between the interdependence and independence of BCAA members' functions.

## Data Availability

The datasets used and analysed during the current study are available from the corresponding author on reasonable request.

## Abbreviations

AKT: Protein kinase B
BCAA: Branched-chain amino acid
BCAT: Branched-chain aminotransferase
BCKDH: Branched-chain alpha-keto dehydrogenase
Cer: Ceramide
DEGs: Differentially expressed genes
Gcg: Proglucagon
Ile: Proglucagon
Leu: Leucine
mTOR: Mammalian target of rapamycin
NF-κB: Nuclear factor-kappa B
PC: Phosphatidylcholine
PE: Phosphatidylethanolamine
TG: Triglycerides
Val: Valine
